# Falling through the worm hole: an exploration of the imaging workup of the vermiform appendix in the pediatric population

**DOI:** 10.1259/bjro.20190016

**Published:** 2019-09-06

**Authors:** Cassandra Sams, Rama S Ayyala, David W. Swenson

**Affiliations:** 1 Department of Diagnostic Imaging, Warren Alpert Medical School of Brown University, Rhode Island Hospital, 593 Eddy St, Providence,

## Abstract

Despite the thousands of articles discussing appendicitis in the literature, the dilemma of how to best diagnosis and manage pediatric appendicitis remains unsettled. Over the past decade, evidence has been mounting about the use of antibiotics as the sole therapy in uncomplicated appendicitis in the adult population. This debate has even recently bled over into the lay press. While this change in practice pattern is still in its infancy for the pediatric population, radiologists should be aware of this change in therapy and how it can impact the imaging work-up and relevant findings. This article concisely summarizes the imaging findings and various imaging pathways to arrive at the diagnose of appendicitis with an emphasis of how to best be of use to our surgical colleagues in this evolving paradigm. It also highlights venues for further research, namely increasing accuracy of differentiating complicated from uncomplicated appendicitis.

## Introduction

For a tiny blind ending tube of hollow viscus, the appendix has garnered quite the reputation. Innumerable articles and debates have centered over this structure whose very function, if it has one, remains shrouded in uncertainty. Most recently, the appendix has even made headlines in the lay press in the USA regarding the utility of appendectomies as a treatment for appendicitis because some believe the medical community may be causing unintended harm by removing this purportedly beneficent organ.^1^

The appendix has been a source of consternation for diagnosticians for at least the last hundred years. Prior to that time, few in the medical community thought this diminutive structure could wreak such havoc within the abdomen.^2^ Confoundingly, the initial symptoms of appendicitis overlap substantially with a myriad of other abdominal and pelvic pathologies, making accurate diagnosis difficult. While urgent appendectomy has been considered, the standard treatment of appendicitis for over a hundred years, a burgeoning school of thought suggests that a more measured approach can be taken with antibiotics as an initial therapy in uncomplicated appendicitis.^3,4^ In fact, there is an active trial underway which pits placebo *vs* antibiotics.^5^ Given the evolving landscape of appendicitis, this article will review the role of radiology, particularly pediatric radiology, in aiding clinicians in their decision making.

### Epidemiology and clinical evaluation

As the most common intra-abdominal surgical emergency, appendicitis affects 7–10% of the population at some point during their life, with the highest incidence seen in the teenage years.^6,7^ Common symptoms of appendicitis are well known. In the most straightforward scenario, a patient will present with periumbilical pain that subsequently migrates to the right lower quadrant. Adjunct symptoms include anorexia, vomiting, and fever. In real world practice, however, the diagnosis is frequently anything but straightforward. The differential diagnosis for right lower quadrant pain is extensive and includes ovarian torsion, omental infarction, pyelonephritis, and gastroenteritis.

The physical exam abounds with eponymous findings including McBurney’s point tenderness (focal tenderness in the right lower quadrant), Rosving’s sign (palpation of the left lower quadrant eliciting pain in the right lower quadrant), Cope psoas sign (pain with hyperextension of the right leg) and the Markle heel drop test (pain elicited by dropping from one’s toes to heels with a jarring landing). However, these clinical findings are notoriously unreliable.^8^ Pediatrics, in particular, has to contend with the difficulties of obtaining a reliable history and physical exam.

Laboratory values, including leukocytosis, elevated polymorphonuclear neutrophils (PMNs) and elevated C-reactive protein (CRP) used in the evaluation of potential appendicitis are all nonspecific markers of inflammation. Therefore their primary utility is ruling out significant pathology when negative.

In order to standardize the approach in the clinical diagnosis of appendicitis, a variety of clinical grading schemes have been described to stratify a patient’s risk of having appendicitis. The Alvarado Scale and Pediatric Appendicitis Scoring (PAS) system are the most frequently used, seen in Tables 1 and 2 respectively. The most commonly described cutoffs for both use a score of 5 or less as not having appendicitis and 6 or 7 and higher as highly associated with appendicitis. In the community setting, with a paucity of dedicated pediatric ER physicians and imagers, these rules are especially valuable to guide the appropriateness of imaging and referral. As a standalone tool, however, they are imperfect. A recent prospective study in the pediatric population (using a cutoff of 7 in both scoring paradigms) demonstrated a sensitivity and specificity of 89 and 59% for the Alvarado score and of 86 and 50% for the PAS system.^6^

**Table 1.** Alvarado scoring system

**Variable**	**Value**
Migration of pain	1
Anorexia	1
Nausea/Vomiting	1
Right lower quadrant tenderness	2
Rebound pain	1
Elevation of temperature ≥37.3°C	1
Leukocytosis ≥10×10^9^/L	2
Polymorphonuclear neutrophilia ≥75%	1

**Table 2.** Pediatric appendicitis scoring system

**Variable**	**Value**
Migration of pain	1
Anorexia	1
Nausea/Vomiting	1
Right lower quadrant tenderness	2
Rebound pain	2
Elevation of temperature ≥38°C	1
Leukocytosis ≥10×10^9^/L	1
Polymorphonuclear neutrophilia ≥75%	1

### Therapeutic interventions

In both the medical and lay press, the increasing use of antibiotic therapy for appendicitis in the adult population is gaining traction.^1,3,4^ The rationale behind changing course after over a century of accepted surgical practice is that complicated (perforated) and uncomplicated (non-perforated) appendicitis are thought to represent two distinct clinical scenarios. Previously, it was thought that uncomplicated appendicitis invariably progressed to complicated appendicitis in the absence of medical intervention. Yet, many recent studies suggest that uncomplicated appendicitis can be managed more conservatively with antibiotics instead of surgery.^9^ While this debate is far from settled, several promising large-scale studies in adults have prompted similar attention in the pediatric population. A meta-analysis by Huang et al analyzed 5 studies, totaling 404 patients, which demonstrated that nonoperative management was successful in 152 of 168 patients (90.5%).^10^ With these promising results, a multicenter randomized control trial is currently underway to further investigate these findings.^7^

Importantly, the use of antibiotics in these studies is limited to “uncomplicated” appendicitis; this classification was defined by most studies in the meta-analysis as symptoms shorter than 48 h, white blood cell count of less than 180,000 /µL, no evidence of pan peritonitis on physical exam, and no evidence of abscess/phlegmon formation on imaging.^10^ The presence of an appendicolith was viewed with concern due to the hypothesis that it may lead to obstruction of the appendiceal lumen, a scenario that would not be addressed by antibiotics alone.

In the ongoing multicenter trial, one of the inclusion criteria is “clinical and or/radiological diagnosis of acute non-perforated appendicitis.”^7^ Thus in this paradigm, not only is the radiologist important in ascertaining the presence of appendicitis but also whether it is perforated. As demonstrated below, the differentiation between these entities on imaging studies remains challenging, warranting continued attention to optimize techniques and refine diagnostic criteria.

## Overview of imaging techniques

### Ultrasound

#### Technique

Before the transducer is laid on the patient, the decision should be made in which patient population should ultrasound be utilized. The easy answer is that any hemodynamically stable pediatric patient is a viable candidate regardless of body habitus or age. The literature bears this out with no weight or BMI limit identified at which an ultrasound should not at least be attempted.^11,12^ No upper (or lower) limits on age exist, with the pediatric literature routinely including patients in their early 20 s.

The basic approach to performing an ultrasound of the appendix has changed little since Puylaert initially described it in the late 1980s.^13^ After asking the patient to localize the pain, a graded compression technique of the right lower quadrant is performed. To clarify a point that is frequently conflated, the graded compression technique does not refer to the compressibility of the appendix, but instead the incremental increase in pressure applied via the transducer with the goal of eliminating the overlying bowel gas. A linear transducer (ranging from 9 to 18 MHz) is the probe most frequently described in the literature, although in the setting of a large body habitus a curvilinear transducer can be employed. Cundy et al reported great success using a tightly curved array transducer, the thought being the small footprint allows greater targeted compression in the area of interest.^14^

**Figure 1.**
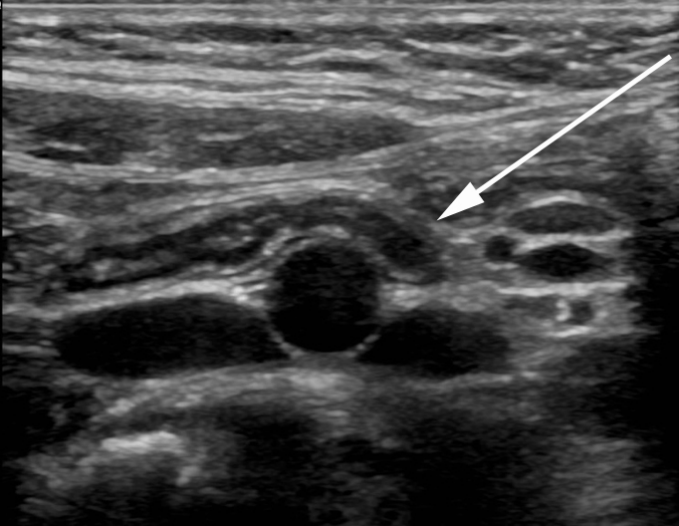
A 8-year-old male with abdominal pain who was diagnosed with gastroenteritis. An image obtained with a 15 MHz linear transducer demonstrates a normal appendix draped over the iliac vessels. The appendix measured 5 mm in diameter (normal), with normal mural architecture and thickness and no increased echogenicity of the periappendiceal fat.

The patient is initially imaged in a supine position. The iliac vessels are a useful and relatively easy anatomic landmark to identify, over which the appendix is frequently draped (Figure 1). In 80% of patients, the appendix is located in this vicinity, either retro-ileal or subcecal.^15^ If the appendix is unable to be visualized with the patient supine, other techniques that can be used are posterior manual compression or rolling the patient in a left lateral decubitus position.

#### Imaging findings

The diagnostic criteria for appendicitis have evolved in the past three decades. Until recently, any appendix with a diameter of greater than 6 mm was considered highly suspicious for appendicitis. However, recent literature suggests that this is too sensitive of a threshold with 7 mm being a more useful cutoff, as close to 40% of otherwise normal appendices measure greater than 6 mm.^16–18^ Regardless of the exact diameter used, having a strict criterion for diameter may be a useful tool but should not be used in isolation to make the diagnosis of appendicitis.

Secondary signs of appendicitis have also been in flux, possibly due to improved transducers allowing better visualization of the appendix and surrounding structures. Initially the presence of an appendicolith was considered suspicious. However, appendicoliths are not uncommonly seen in a normal appendix, a fact that was even noted in the original description of appendicitis.^2^ Thus in the absence of other concerning features, an appendicolith is not a source of alarm with a recent study noting that (adult) patients with incidentally discovered appendicoliths were at no higher risk of developing appendicitis than the general population.^19^ When there is high suspicion for appendicitis, however, an appendicolith should be reported as its presence has the ability to change management of the patient.^10^

The degree of compressibility has also fallen out of favor as a number of factors are at play that reduce the consistency of this finding, namely body habitus, the pressure applied by the sonographer, and the location of the appendix.^16^ The degree of hyperemia is similarly subject to the pitfalls of technique and subjective determination of what constitutes increased blood flow.^15,16^

**Figure 2.**
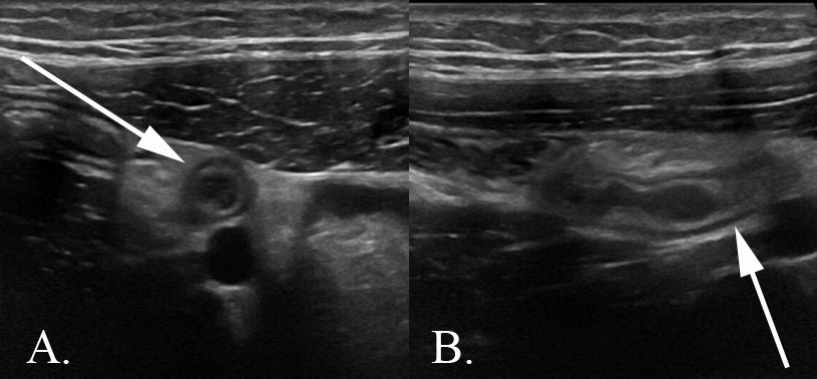
A 14-year-old male with abdominal pain who was diagnosed with uncomplicated appendicitis. Images obtained with a 15 Hz linear transducer. (A) The appendix in short axis and (B) in long axis is enlarged, measuring 8 mm in diameter. The surrounding periappendiceal fat demonstrates increased echogenicity with subtle mass effect on the overlying abdominal musculature.

Although not initially described, increased echogenicity of the periappendiceal fat has been demonstrated to a useful secondary signs (Figure 2).^17,20^ The loss of mural stratification has been shown repeatedly to be a helpful adjunct, with apparent discontinuity of the wall increasing the likelihood of perforated appendicitis. Changes to the adjacent bowel (loss of normal peristalsis, thickening) are also useful with the caveat that the primary inflammatory process can actually be the bowel with secondary inflammatory changes of the appendix.

**Figure 3.**
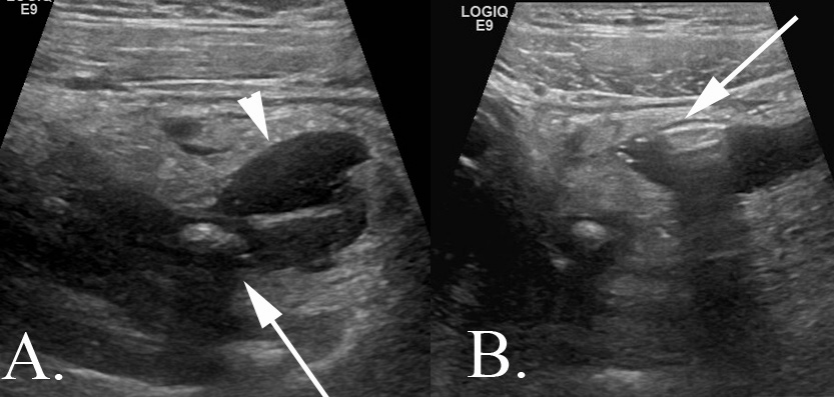
A 14-year-old male with abdominal pain diagnosed with complicated appendicitis. Images obtained with a 9 MHz linear probe. (A) An arrow indicates the shadowing appendicolith contained with a thickened appendix. The arrowhead demonstrates a small pocket of complex fluid. It is important to note in the report whether or not the appendicolith is intraluminal or contained within the abscess as it can serve as a nidus of recurrent infection. (B) The arrow demonstrates free air casting a “dirty shadow” within the pocket of complex fluid. Both images show marked echogenicity of the surrounding periappendiceal fat.

Small volume, simple free fluid is frequently seen in the setting of a normal appendix.^15,17,20^ However, the presence of a large amount of fluid, particularly when it is complex, remains helpful as does the presence of a frank abscess. In the setting of other secondary findings, the presence of complex fluid should be reported as highly suspicious for complicated appendicitis (Figure 3).^21,22^ The presence of free air on ultrasound is also pathognomonic for perforation, although small amounts can be challenging to appreciate. Unfortunately, the sensitivity for the detection of perforation on ultrasound is unreliable, ranging from 29 to 84%.^40,41^ The sonographic findings of acute appendicitis are summarized in Table 3.

**Table 3.** Major imaging findings for uncomplicated and complicated appendicitisModality

	Uncomplicated	Complicated
Ultrasound	Enlarged appendix (>6–8 mm), mural thickening, increased echogenicity of the periappendiceal fat, complex free fluid^17–20^	Abscess formation, frank mural discontinuity, extraluminal appendicolith, free air (although frequently challenging to detect on us)^21,22^
CT	Enlarged appendix (>6–8 mm), mural thickening, hyperenhancement of the wall, stranding of the surrounding mesenteric fat^23–25^ ^25–28^ ^29^ ^30^ ^31^ ^32^	Abscess formation, frank mural discontinuity, extraluminal appendicolith, free air^33^ ^34^ ^35^
MRI	Enlarged appendix (>7–8 mm), mural thickening, edema of the wall of the appendix and of the surrounding mesenteric fat^36^ ^37^ ^38^ ^39^	Abscess formation, frank mural discontinuity, extraluminal appendicolith, free air (although both free air and an extraluminal appendicolith can be challenging to detect on MR)

With this arsenal of sonographic findings, a positive exam holds great clinical weight as does a negative exam with a fully visualized appendix. Conversely, a radiologist can also readily determine when an ultrasound is indeterminate and recommend advanced imaging for definitive evaluation. The challenge arises when the appendix is not visualized or only partially visualized. The general trend is that non-visualization of the appendix in the absence of secondary signs can be considered negative.^42,43^ But this is certainly not unanimous with recent studies showing a lack of sensitivity based solely on the secondary signs in the setting of a non-visualized appendix.^44,45^

#### Computed tomography

##### Technique

During the 1990s, abdominopelvic CT became integral to the imaging work-up of pediatric patients with suspected appendicitis.^23,46–50^ Over the past 30 years, both imaging protocols and CT technology have evolved.^51,52^ More recently in response to the Image Gently^®^ campaign, efforts to optimize appendix CT have focused on limiting its use to patients with equivocal ultrasound exams,^53^ and reducing radiation doses while maintaining diagnostic efficacy.^54–57^

Despite decades of research and routine clinical use, there remains no universally accepted protocol for CT of suspected appendicitis.^58^ Various authors have shown extremely high sensitivities and specificities with a variety of techniques: using only rectal contrast^59^; using only i.v. contrast^60^; and even no oral, rectal, or i.v. contrast.^61^ With this last technique, the authors acknowledged that several of their false positives and false negatives were young patients with little intra-abdominal fat, highlighting the potential benefits of contrast material for evaluating pediatric appendicitis. In general, the most widely accepted CT protocols include both i.v. and oral contrast material.^58^

As mentioned above, concerns over the risks of ionizing radiation in children have led national organizations to encourage reductions in CT utilization when alternative imaging modalities can provide the same diagnostic information. Recently, a study of 45 pediatric hospitals in the USA showed there is a transition away from CT use for appendicitis: in 2005, 59.1% of children with appendicitis had a CT while only 25% had an ultrasound; in contrast in 2014, 32.7% had a CT and 61% had an ultrasound.^62^

When CT is used, dose reduction techniques should be employed to minimize radiation exposure while maintaining diagnostic utility. In 2015, Callahan et al reported that helical CT using a standard reconstruction algorithm could be performed at 50% dose, resulting in similar rates of appendix visualization^55^ and similar diagnostic yield of CT in clinical practice.^54^ Didier et al reported that using iterative reconstruction methods reduced CT radiation dose by 45% without compromising diagnostic accuracy.^57^ Updating CT protocols to reflect up-to-date dose reduction techniques, and adopting a stepwise Ultrasound-CT imaging pathway may represent the greatest current opportunity for imaging centers to implement quality improvement in their practices.^63–69^

##### Imaging findings

**Figure 4.**
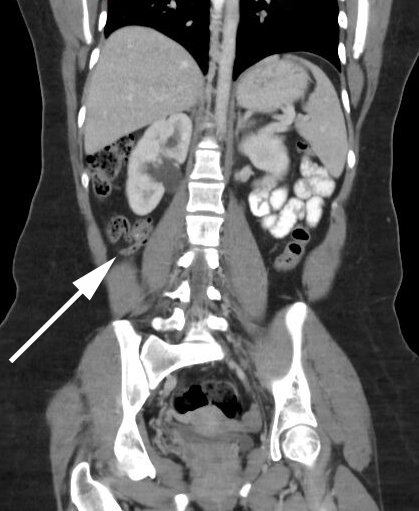
A 16-year-old female with abdominal mass (not pictured). A CT coronal image demonstrates a normal caliber appendix, coiled in the right mid abdomen. There is no evidence of mural thickening. The surrounding periappendiceal fat is pristine, and there is intraluminal gas. Incidentally noted is mild right sided urinary tract dilatation.

The most commonly described CT finding of appendicitis is dilation, however, the definition of appendiceal dilation has been inconsistent. Two recent articles provide valuable normative data for the pediatric appendix,^24,26^ noting that appendiceal diameters are normally distributed, with 95% confidence interval including diameters up to 8.7 mm (Figure 4).^26^ These data highlight that appendiceal diameter alone is not enough to confirm a diagnosis of appendicitis on CT.

**Figure 5.**
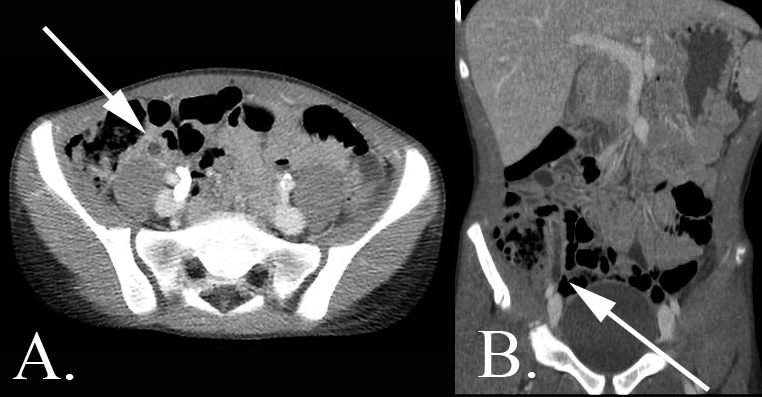
A 13-year-old male with abdominal pain who was diagnosed with uncomplicated appendicitis. (A) Axial CT and (B) coronal CT images demonstrates a dilated fluid filled appendix with a thickened and mildly hyperenhancing appendiceal wall. This appendix has a taut, elongated morphology as opposed to the redundant and coiled appearance of the normal appendix. The paucity of intraabdominal fat in this child makes the assessment of periappendiceal fat stranding challenging.

Additional suggestive findings of appendicitis include: mural thickening and increased enhancement; presence of an obstructing appendicolith^23,25,27,28^; evidence of periappendiceal inflammation, including fat stranding, fascial thickening, cecal wall thickening; and presence of free fluid within the right lower quadrant or deep pelvis (Figure 5).^23,25,27–32^ Importantly in the absence of such findings, CT can be interpreted as negative for appendicitis with a negative predictive value of 98.7%, even if the appendix is not visualized.^51^

**Figure 6.**
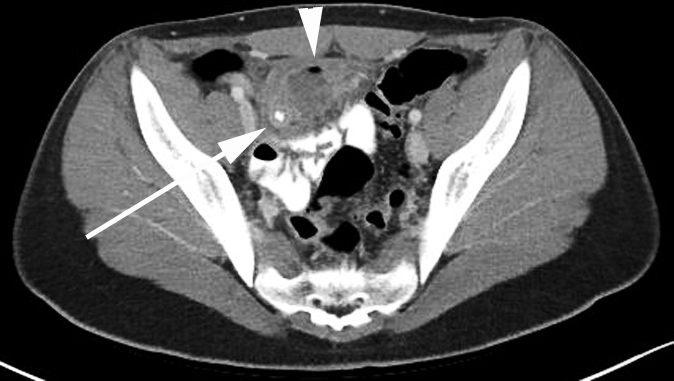
A 14-year-old male with abdominal pain who was diagnosed with complicated appendicitis (same patient as Figure 3). Axial CT image demonstrates an appendicolith within the lumen of the dilated appendix. There is a surrounding air and fluid containing collection consistent with an abscess.

Specific imaging features that suggest perforation include focal defect in the enhancing appendiceal wall, extraluminal air, extraluminal appendicolith, prominent periappendiceal inflammation or phlegmon, and abscess (Figure 6).^33,34^ However, these findings are not particularly sensitive for perforation.^33,34^ In a multireader analysis of 200 patients, reviewers had an overall sensitivity of 62% and specificity of 81% for determining perforation by CT.^70^ Thus, despite the variability in referenced criteria for differentiating uncomplicated and complicated appendicitis on CT, the overall performance remains excellent with sensitivities in recent studies ranging from 94 to 95% and specificities of 92 to 95%.^35,71^ Table 3 summarizes the CT findings of acute appendicitis.

##### Magnetic resonance (MR)

###### Technique

In recent years, MR has emerged as a viable modality to assess for appendicitis in children. This growing utilization has been in part due to increasing awareness of potential long-term effects of radiation associated with CT in childhood.^72,73^

Review of the literature illustrates the wide variety of MR protocols used in the evaluation for appendicitis. A commonality found in all protocols is the inclusion of *T_2_* weighted single shot fast spin echo sequences, both with and without fat saturation, usually in the axial and coronal planes.^74^ Some authors have shown success with these sequences alone,^75,76^ while other studies have shown advantages of including additional sequences. Diffusion-weighted imaging in particular has been shown to be helpful in detecting restricted diffusion in the appendiceal wall^77–79^ as well as increasing the conspicuity of potential abscesses.^80^ As is frequently the case with MR, a balance must be struck between the utility of adding sequences and the length of the study.

Use of i.v. gadolinium-based contrast for evaluation of appendicitis has been controversial, particularly given current safety concerns regarding the potential for deposition of free gadolinium in the brain and soft tissues.^81^ Several studies have shown similar effectiveness between the non-contrast enhanced protocols and the protocols using gadolinium enhanced *T_1_* weighted images (in addition to the *T_2_* weighted imaging).^82–84^ Given the concern over gadolinium exposure, non-contrast enhanced MR is preferable as it is both a safe and diagnostic option for evaluation of pediatric appendicitis.

In general, MR examinations are lengthy. This creates the potential problem of patient motion, a problem potentiated in the pediatric population. Sedated exams can be performed to combat this challenge. However, sedation is associated with inherent risks, such as aspiration and respiratory depression, as well as increased cost and personnel demands.^85,86^ Particularly in the younger patient population (<2 years of age), there is increasing reticence to use sedation.^87^ An additional pragmatic consideration is that sedated exams can tie up the scanner for longer periods of time, a fact which may be untenable in a busy department. However, studies have shown success utilizing non-contrast-enhanced MRI in pediatric patients as young as 4 years old without sedation, especially with emerging faster, shorter protocols.^76^

###### Imaging findings

**Figure 7.**
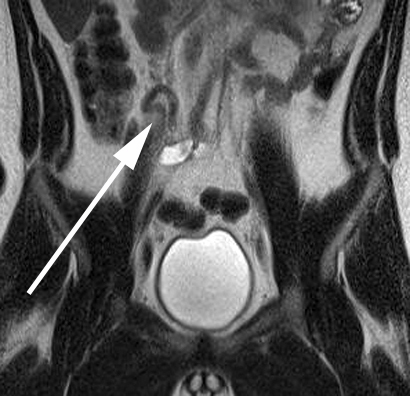
A 17-year-old male with abdominal pain. Coronal *T_2_* weighted MR image without fat saturation demonstrates a normal caliber appendix adjacent to the cecum, draping over the iliac vessels. No fluid or edema is seen surrounding the appendix, and there is no evidence of mural thickening. As on the CT image of the normal appendix, it is coiled upon itself as opposed to the taut morphology seen in an inflamed appendix.

**Figure 8.**
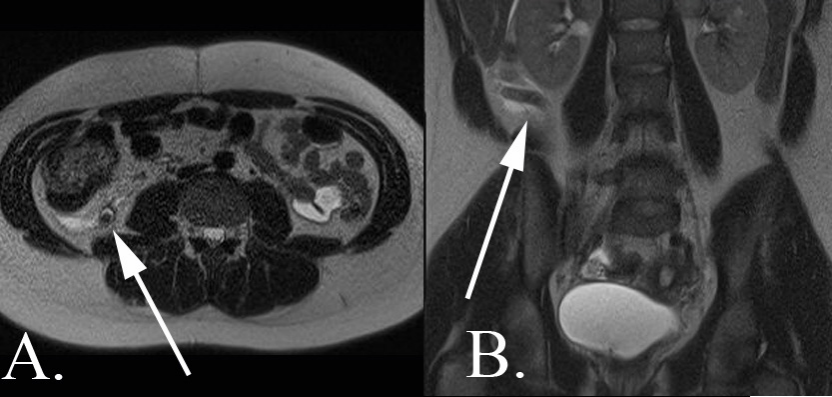
A 14-year-old female with abdominal pain who was diagnosed with acute uncomplicated appendicitis. (A) Axial *T*_2_ weighted MR image without fat saturation demonstrates a dilated appendix with surrounding fluid and edema of the mesenteric fat. Within the appendiceal lumen, there is a round T2 hypointense structure consistent with an appendicolith. (B) Coronal *T_2_* weighted MR image without fat saturation again shows the dilated appendix with surrounding small volume free fluid.

**Figure 9.**
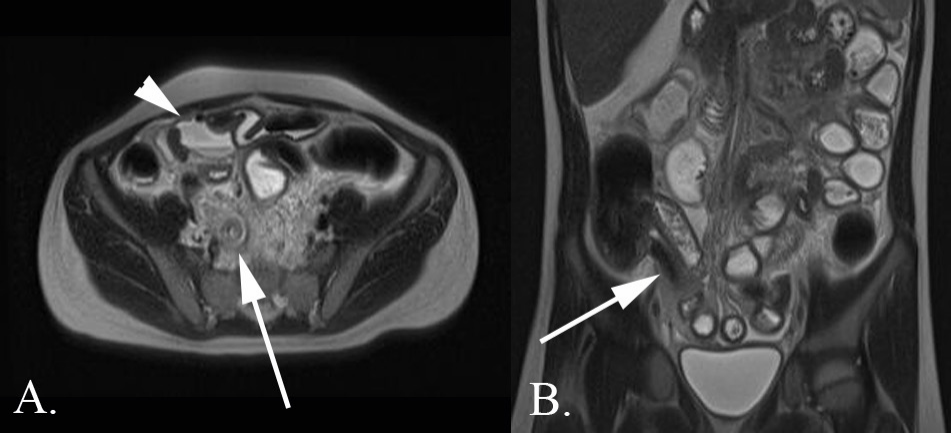
A 9-year-old male with abdominal pain who was diagnosed with complicated appendicitis. (A) Axial *T_2_* weighted MR image without fat saturation demonstrates a markedly dilated appendix (arrow) with pronounced inflammatory changes of the periappendiceal fat. There is a complex fluid collection containing a fluid*–*fluid level and a locule of free air (arrowhead). (B) Coronal *T_2_* weighted MR image without fat saturation again demonstrates a markedly dilated appendix. Small volume free fluid is noted throughout the abdomen with dilated loops of small bowel consistent with developing small bowel obstruction.

The normal appendiceal diameter on MR has been shown to average 5–7 mm (Figure 7).^36^ Dieder et al showed that a diameter greater than 7 mm and appendiceal wall thickness greater than 2 mm to have good predictive power for acute appendicitis.^37^ As with ultrasound and CT, an enlarged appendiceal diameter cannot be used as sole criteria for appendicitis on MR, but rather needs to be used in context with additional findings to make accurate diagnosis.^74^ Other positive prognostic factors for appendicitis include focal periappendiceal inflammation and fluid, manifested by increased T2 signal in the surrounding fat; an appendicolith, manifested by a focus of diminished signal intensity on all sequences; and abscess formation, manifested by T2 hyperintense collection with a wall (Figure 8). Destruction of the appendiceal wall can be seen in both perforated and nonperforated appendicitis.^38^ Free air is pathognomonic for perforation but is rarely seen with routine MR sequences (Figure 9).^38^ Table 3 presents a concise description of the MR findings in acute appendicitis.

As with the prior modalities, the distinction between perforated and non-perforated appendicitis is challenging. While Dillman et al demonstrated a sensitivity of 90% and specificity of 99% for the evaluation of perforation utilizing MR,^75^ the results of other studies are not nearly as encouraging. In the adult population, Leewenburgh et al described a sensitivity of 57% and specificity of 86%.^38^ I.v. contrast does not play a specific role in this distinction, although peritoneal enhancement may help with determining perforation.^79^

###### Imaging algorithm

For the “appropriate” work-up of appendicitis, there is not a one size fits all approach. The imaging algorithm will vary depending on the resources available at a specific institution. Ultrasound is a reasonable first line imaging modality. However, institutions who do not perform routine pediatric imaging may feel uncomfortable with both performing and interpreting appendicitis ultrasound. There is a relatively steep learning curve in both of these aspects, and, if the volume at a particular facility is not sufficient, it can be challenging to maintain an adequate level of competency. Even in an era of hypervigilance regarding radiation, if it is not feasible to make the correct diagnosis with ultrasound, there should be no hesitancy in proceeding to CT (or MR, as available). Additionally, the work flow of an institution, particularly if patient through put is paramount, may make ultrasound an untenable solution as the exam itself can be lengthy relative to CT. Moreover, while not a requisite of appendicitis ultrasound *per se*, a full bladder in the pediatric female population is desirable to exclude pelvic pathology mimicking appendicitis, which may further increase the length of time associated with the exam.

MR is the newest imaging modality in the assessment of appendicitis thus many of the “kinks” in perfecting it for this application are still being worked out. Additionally, body MR, and pediatric body MR specifically, is not necessarily widely read by general radiologists. There is also the very real limitation of scanner accessibility. However, if an institution has adequate MR access, the protocols are relatively straightforward to implement. In addition, compared to ultrasound, the learning curve for image acquisition and interpretation for MR is arguably not as steep. A recent study by Covelli et al demonstrated the feasibility of implementing a pediatric appendicitis MR protocol in a general adult hospital with non-pediatric radiologists, emergency physicians, and surgeons.^39^ While this is a single study, it lays the foundation for pediatric appendicitis MR to move from academic centers into community hospitals. Another study published earlier this year explored the utility of using MR as a first line imaging modality in children with suspected appendicitis.^88^ Given its lack of ionizing radiation, feasibility without i.v. contrast, and relative operator independence (as well as its exquisite soft tissue contrast to aid in the detection of other intra-abdominal pathology), MRI may very well supplant CT and ultrasound as the first line imaging choice for suspected appendicitis in the future.

Finally, there is CT. This imaging modality has been the lynchpin for the diagnosis of appendicitis for the past three decades, particularly in the adult population. While the paucity of intraabdominal fat in the younger patients can increase the level of difficulty in interpretation, CT has three big advantages: it is readily available, quick to obtain, and highly specific and sensitive for the diagnosis in the hands of both general radiologists and pediatric subspecialists. Thus, even if CT is the only imaging modality available, the diagnosis can be made quickly and correctly, which is ultimately what is most important for patient care.

In a scenario with a full complement of resources available, a reasonable work-up of appendicitis begins with ultrasound. For equivocal or indeterminate cases, this may be followed by a non-contrast enhanced MR CT can be used as a problem-solving tool on an as needed basis.

###### Standardized reporting schema

Regardless of the imaging modality, it is of utmost importance that the radiology report conveys the relevant findings correctly and concisely. A number of studies have demonstrated that standardizing the reporting format across radiologists not only increased clinician satisfaction but impacts the accuracy of reports.^89,90^ In the age of report templates, this should be relatively easy to implement and share within a group.

In an ideal world, parsing out the individual imaging findings in a granular format and codifying this across institutions would make a powerful research tool. With the ever expanding world of artificial intelligence and clinical decision support, it is possible that specific imaging features in conjunction with specific clinical findings, could be combined in yet undetermined ways to not only ascertain the presence of appendicitis but also assess the likelihood of perforation.

## Conclusion

The primary role of imaging in the work-up of appendicitis now and in the immediate future is to determine the presence of appendicitis. As described above, each of the imaging modalities can readily arrive at the diagnosis and which modality to choose depends on the resources at one’s institution. In an experienced hand and an ideal patient, ultrasound can provide unparalleled resolution of the appendix. Conversely, ultrasound can also fall short of a diagnostic exam. Avoiding the radiation associated with CT or the expense associated with MRI can be admirable goals. However, they should not deter the radiologist or clinician from pursuing a definitive diagnosis. In the end the best test is the one that provides the patient with an answer.

As the debate over the appropriateness of antibiotics as the primary therapy of appendicitis continues, radiologists have an opportunity to play an important role in differentiating complicated from uncomplicated appendicitis. Yet, according to currently available data, no modality is adequate for accurately assessing the presence of appendiceal perforation. However, this distinction may not be a reliable indicator of the true clinical picture. All perforations are not created equal; missing a microperforation found at the appendiceal tip by the pathologists likely does not have the same clinical ramifications as missing frank purulence found at the time of surgery. Arguably, the former could still be considered “uncomplicated appendicitis.” While some studies have relied on the criteria of the presence of phlegmon, abscess, and/or signs of peritonitis for the diagnosis of complicated appendicitis,^10^ other research, most notably the ongoing multicenter clinical trial, equates perforated appendicitis with complicated appendicitis.^7^ The distinction between these two entities needs to be formally elucidated with the help of clinical colleagues and then renewed focus can be paid to what imaging features differentiates complicated *vs* uncomplicated appendicitis.

Over the past century and a half, our understanding of appendicitis has dramatically evolved. From not even recognizing it as a common instigator of abdominopelvic pathology to accepting a 20% false-positive rate at the time of appendectomy to exploring the possibility of a nonsurgical approach to therapy, radiology has been and will continue to be integral in this transformation.
